# Healthcare factors associated with the risk of antepartum and intrapartum stillbirth in migrants in Western Australia (2005-2013): A retrospective cohort study

**DOI:** 10.1371/journal.pmed.1003061

**Published:** 2020-03-17

**Authors:** Maryam Mozooni, Craig E. Pennell, David B. Preen

**Affiliations:** 1 School of Population and Global Health, The University of Western Australia, Perth, Western Australia, Australia; 2 School of Medicine and Public Health, The University of Newcastle, Callaghan, New South Wales, Australia; Columbia University Mailman School of Public Health, UNITED STATES

## Abstract

**Background:**

Migrant women, especially from Indian and African ethnicity, have a higher risk of stillbirth than native-born populations in high-income countries. Differential access or timing of ANC and the uptake of other services may play a role. We investigated the pattern of healthcare utilisation among migrant women and its relationship with the risk of stillbirth (SB)—antepartum stillbirth (AnteSB) and intrapartum stillbirth (IntraSB)—in Western Australia (WA).

**Methods and findings:**

A retrospective cohort study using de-identified linked data from perinatal, birth, death, hospital, and birth defects registrations through the WA Data Linkage System was undertaken. All (*N* = 260,997) non-Indigenous births (2005–2013) were included. Logistic regression analysis was used to estimate odds ratios and 95% CI for AnteSB and IntraSB comparing migrant women from white, Asian, Indian, African, Māori, and ‘other’ ethnicities with Australian-born women controlling for risk factors and potential healthcare-related covariates. Of all the births, 66.1% were to Australian-born and 33.9% to migrant women. The mean age (years) was 29.5 among the Australian-born and 30.5 among the migrant mothers. For parity, 42.3% of Australian-born women, 58.2% of Indian women, and 29.3% of African women were nulliparous. Only 5.3% of Māori and 9.2% of African migrants had private health insurance in contrast to 43.1% of Australian-born women. Among Australian-born women, 14% had smoked in pregnancy whereas only 0.7% and 1.9% of migrants from Indian and African backgrounds, respectively, had smoked in pregnancy. The odds of AnteSB was elevated in African (odds ratio [OR] 2.22, 95% CI 1.48–2.13, *P* < 0.001), Indian (OR 1.64, 95% CI 1.13–2.44, *P* = 0.013), and other women (OR 1.46, 95% CI 1.07–1.97, *P* = 0.016) whereas IntraSB was higher in African (OR 5.24, 95% CI 3.22–8.54, *P* < 0.001) and ‘other’ women (OR 2.18, 95% CI 1.35–3.54, *P* = 0.002) compared with Australian-born women. When migrants were stratified by timing of first antenatal visit, the odds of AnteSB was exclusively increased in those who commenced ANC later than 14 weeks gestation in women from Indian (OR 2.16, 95% CI 1.18–3.95, *P* = 0.013), Māori (OR 3.03, 95% CI 1.43–6.45, *P* = 0.004), and ‘other’ (OR 2.19, 95% CI 1.34–3.58, *P* = 0.002) ethnicities. With midwife-only intrapartum care, the odds of IntraSB for viable births in African and ‘other’ migrants (combined) were more than 3 times that of Australian-born women (OR 3.43, 95% CI 1.28–9.19, *P* = 0.014); however, with multidisciplinary intrapartum care, the odds were similar to that of Australian-born group (OR 1.34, 95% CI 0.30–5.98, *P* = 0.695). Compared with Australian-born women, migrant women who utilised interpreter services had a lower risk of SB (OR 0.51, 95% CI 0.27–0.96, *P* = 0.035); those who did not utilise interpreters had a higher risk of SB (OR 1.20, 95% CI 1.07–1.35, *P* < 0.001). Covariates partially available in the data set comprised the main limitation of the study.

**Conclusion:**

Late commencement of ANC, underutilisation of interpreter services, and midwife-only intrapartum care are associated with increased risk of SB in migrant women. Education to improve early engagement with ANC, better uptake of interpreter services, and the provision of multidisciplinary-team intrapartum care to women specifically from African and ‘other’ backgrounds may reduce the risk of SB in migrants.

## Abstract 2

Maryam Mozooni and colleagues reveal the higher risk of intrapartum stillbirth for migrant women in Australia compared to Australian born women.

## Author summary

**Why was this study done?:**

- Despite the availability of advanced pregnancy and childbirth care, the rate of stillbirth (SB) is unacceptably high among ethnic groups and migrant populations living in high-income countries.
- Nonwhite ethnic and migrant groups, especially those from African and other non-English speaking backgrounds, have a higher risk of SB compared with white and/or nonmigrant populations.
- Known risk factors do not explain the observed increased risk, and more investigation is needed to identify influential factors specific to those populations.

**What did the researchers do and find?:**

- Using routinely collected administrative health and registry data, this study investigated all births to the non-Indigenous population of Western Australia (WA) from 2005 to 2013, including 260,997 live births and SBs.
- Health-service related factors were investigated to identify the pattern of service utilisation that may contribute to the increased rate of SBs in at-risk populations.
- Late commencement of ANC, not utilised interpreter services, lack of private health insurance, and midwife-only care during birth were associated with increased risk of SB in specific ethnic groups of migrants.

**What do these findings mean?:**

- Engaging women with ANC early in pregnancy, offering interpreter service proactively, providing more frequent ultrasound surveillance, and involving a team (both doctor and midwife) for care during birth for specific at-risk groups may reduce the risk of SB.
- A culturally responsive health system can meet the educational and healthcare needs of at-risk populations.
- Cautious interpretation of findings is recommended. Enhanced data records on obesity, ANC, and labour care information can strengthen studies like this even further to inform policy and practice.

## Introduction

Despite the availability of quality antenatal and obstetric care in most developed nations, disparities in rates of stillbirth (SB) within and between countries continue to be reported [1,2]. Where similar health systems exist, different ethnic composition may explain some of this variation between countries [3].

In Australia, migrant women are at increased risk of SB compared with Australian-born women, despite having access to the same health resources [4–6]. Specifically, we observed an increased rate of antepartum stillbirth (AnteSB) in migrant women from African, Indian, and ‘other’ nonwhite ethnic backgrounds [6]. Further, we reported an increased rate of intrapartum stillbirth (IntraSB) in African and ‘other’ nonwhite migrant ethnicities despite adjusting for several well-established risk factors for SB [6]. This warranted an investigation for additional factors that may explain the higher risk of SB in migrant populations. Targeting such specific factors in these at-risk populations is imperative for evidence-based practice and a precise public health plan for reducing risk of SB in migrants. Evidence suggests that the risk profile of migrants differs from that of the native-born population [6,7], and strategies that are found effective in their native-born counterparts, such as lowering alcohol or tobacco consumption in pregnancy, may not yield similar effect on a population level because of a considerably low prevalence of those habits among them [6,7]. In contrast, communication barriers [8] and factors such as access to or timing of ANC [7,8] and uptake of other services, because of unfamiliarity or socioeconomic or private health insurance status, may play a greater role in these groups [7, 9,10].

Thus, we hypothesised that healthcare factors may explain the disparities observed in the risk of SB between migrant and Australian-born populations. We investigated whether pattern of healthcare utilisation among migrant women in Western Australia (WA) is different to that of the Australian-born population and if such difference influences the risk of SB. We specifically investigated the relationship between SB and timing of ANC, utilising interpreter services, health insurance status, and type of intrapartum care.

## Methods

### Study design and participants

A retrospective cohort study using routinely collected administrative health data was undertaken. We examined de-identified data for the entire non-indigenous population of births occurred in WA from 1 January 2005 to 31 December 2013 through the WA Data Linkage System (WADLS) of WA Department of Health. No separate protocol for this study is available other than the previous study published from the same project [6].

### Data sources and linkage

WADLS was formally established in 1995 as a collaboration between the WA Department of Health and researchers, mainly for population health research purposes. It has a highly successful history of linking data, dating back to the 1970s, and in just its first 10 years of operation has supported more than 400 studies contributed to policy, practice, and wellbeing of the population [11].

WADLS applies probabilistic matching based on full name and address, phonetic compression algorithms, and other identifiers to link data from a variety of health and other administrative data sets [12]. The frequency of invalid or missed links based on evaluation of linked chains is estimated to be very low (0.11%), and the linkage procedures are widely known as best practice [11–13].

Data for this study was accessed from multiple data collections. We primarily utilised the Midwives Notification System (MNS), a statutory highly reliable data collection of demographic, pregnancy, and delivery information for all births in WA. The MNS adheres to strict quality assurance processes ensuring data completeness, validity, and reporting compliance [14]. To supplement MNS data, other WA statutory data collections—the Hospital Morbidity Data Collection (HMDC) containing data related to inpatient discharges from all private and public hospitals [15], the WA Registry of Developmental Anomalies (WARDA) containing a database of developmental anomalies identified by 6 years of age, including fetuses of terminated pregnancies [16], and Birth and Death Registrations [17]—were used. These data collections are key information sources to meet the mandatory and statutory reporting requirements in WA. Further, genealogical linkage through the Family Connections Linkage Facility of the WADLS was used to link women with child outcomes [18].

**Fig 1:**
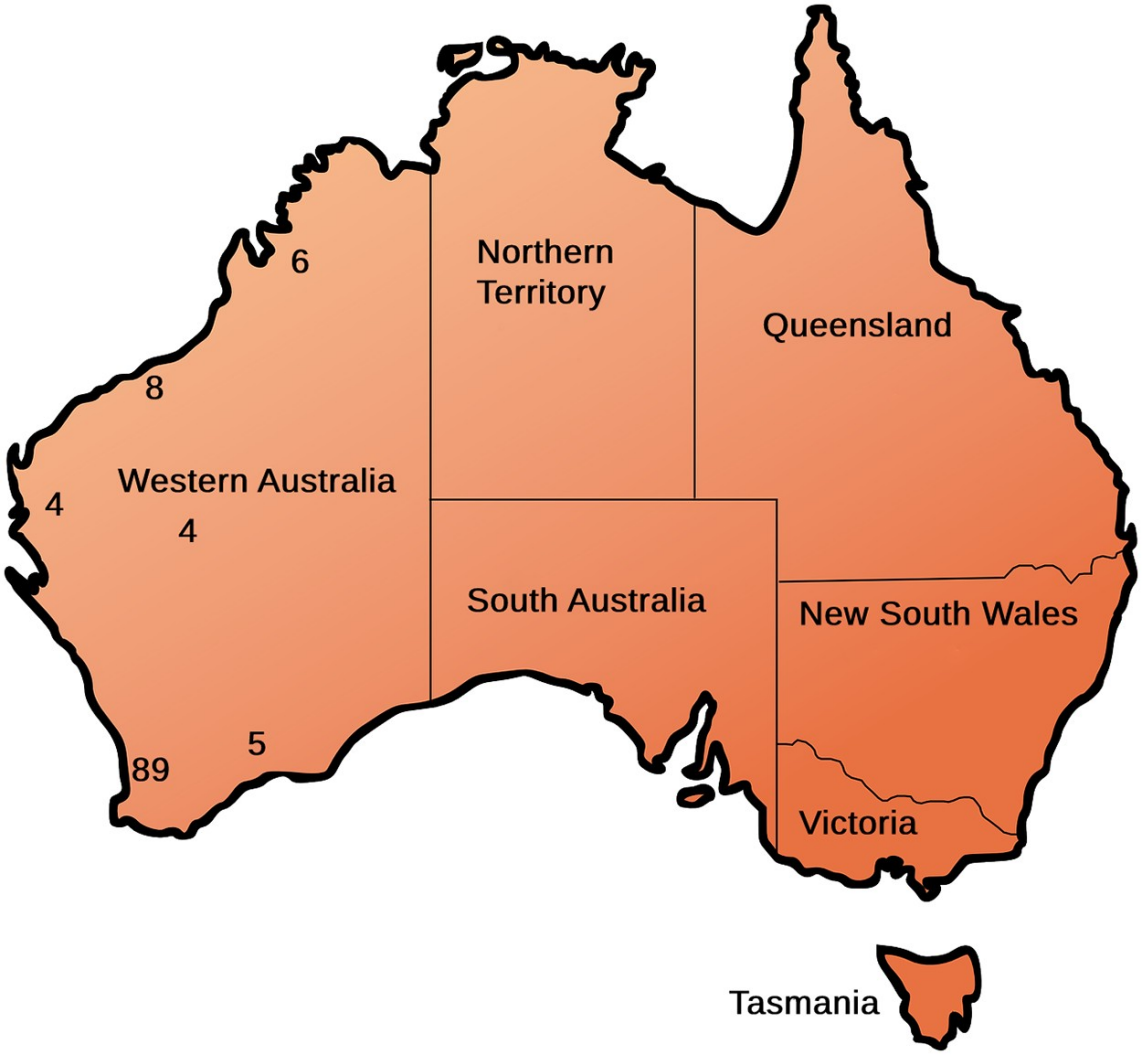
Geographical location of the private and public hospitals in WA. Base image by OpenClipart-Vectors from Pixabay.

Fig 1 shows the geographical location, the number, and the distribution of hospitals across WA [19]. Additional information can be found in the S1 WA Health System.

### Exposures

Migrant status for mothers, defined as country of birth other than Australia, was ascertained through mother’s place of birth variable from Birth Registration data or country of birth from mother’s HMDC records. Mother’s place of birth variable included city, province, and country of birth as reported by parents on the Birth Registration Form. Utilising this variable, a new variable was created to classify the population as Australian-born or migrant. Place of birth from Birth Registration data was merged with MNS data, which was complete for 99.0% of the births. Country of birth from the mother’s hospital record was used to ascertain the mother’s place of birth and to retrieve the missing values; thus, maternal migrant status achieved 99.99% completeness for the population of study.

The migrant population was further stratified by self-reported ethnicity using the variable ethnic origin, which was 100% complete (MNS data), as white (Caucasian), Asian, Indian, African, Māori, and ‘other’, and was compared with Australian-born women from any ethnicity. For this comparison, a categorical variable with values assigned as Australian-born, white, Asian, Indian, African, Māori, and ‘other’ was created. We did not stratify Australian-born population by ethnicity because we had previously reported that the proportion of nonwhite Australian-born women was very small (3.3%) and the risk of SB in them was similar to that of white Australian-born women [6], with no SBs occurring in Australian-born women from Indian or African backgrounds.

Because the focus of this project was on migrants, any data for women of Aboriginal or Torres Strait Islander background were excluded by design. This is also an approach to eliminate the risk of nondifferential misclassification bias toward the null hypothesis [5,6] because the prevalence of SB among this population is twice that of non-indigenous Australians [20].

### Outcomes

According to the Australian Institute of Health and Welfare and the National Perinatal Data Collection, SB is defined as death of a baby of at least 20 completed weeks of gestation or 400 g or more birth weight if the gestation was unknown, before the complete expulsion or extraction from its mother [21]. We further categorised SB as antepartum (death before commencement of labour) or intrapartum (death after labour started) [1]. Data on type of SB was available for 99.98% of SBs by cross-source checking of status of baby at birth (MNS) and presence or absence of the fetal heart beat at the commencement of labour (death certificates). Any termination of pregnancy, identified through WARDA and death records, were excluded (*n* = 433).

### Other variables

Hospital type (tertiary, metro-public, metro-private, and rural-private/public), interpreter use (yes/no)—indicating if an official paid interpreter service was used, and private health insurance status (yes/no)—indicating whether the patient had hospital insurance—were available for all hospital births (99.0%). The 2 variables, interpreter use and health insurance status, were used to investigate the potential influences of access to healthcare services and communication barriers on the risk of SB in ethnic migrant groups.

Data on intrapartum care provider were reported as accoucheur (birth attendant/supervisor) on MNS and was available for all births—coded as obstetrician, other medical practitioner, midwife, student, self/no attendant, and other; each case could contain single or multiple values. For analyses, this variable was categorised as midwife, doctor, mix (team), and self/no care. Also, a dichotomous variable, midwife-only (yes/no), was created.

Gestational age at first antenatal care (ANC) visit, recorded in MNS since 2010, was used to stratify the population (2010–2013) by timing of commencement of ANC (late booking: first visit after week 14) [10,22] in a subgroup analysis.

Low birth weight (LBW), defined as birth weight <2,500 g [23], and preterm birth (PTB), defined as birth before 37 weeks’ gestation [24], are considered intermediate variables and not confounders, and adjusting for them in perinatal mortality analyses can create bias [25]. Therefore, instead of adjusting for these 2 variables, adjusting for their risk is suggested [25,26]. Hence, the predicted probability of LBW or PTB above the 95th percentile [26] titled ‘high-risk-of-LBW’ and ‘high-risk-of-PTB’ were calculated and used in the analyses instead.

The Index of Relative Socioeconomic Disadvantage (IRSD), summarising several disadvantage measures, including low income, low education, high unemployment, and unskilled occupations [27], and Accessibility/Remoteness Index of Australia (ARIA) [28] were derived and provided by geocoding using address-parsing software by WADLS. ARIA is a geographical index defining remoteness based on accessibility to goods, services, and opportunities for social interaction across Australia based on road distance from populated towns [28]. A missing subgroup was created for ARIA and IRSD for missing data (3.3%) to keep all the cases in the analysis.

### Statistical analysis

Demographic and obstetric characteristics of study groups were tabulated. Pearson χ^2^ or Fisher exact tests were used as appropriate for descriptive analyses. Independent samples *t* test was used to compare means for continuous variables. Cumulative incidence rates of SB (overall, AnteSB, IntraSB), stratified by ethnicity, were calculated over the period of study, with denominators determined by 10,000 total births (live and SB).

Univariable logistic regression analysis for the whole population of study was used to examine association between risk factors and each type of SB and to calculate crude odds ratio (OR) and 95% CI. Multivariable logistic regression for all analyses included established (ethnicity [4–6,29–35], year of birth [6,32,33,35–39], marital status [6,39–41], maternal age group [5,6,32,37–39,42], parity [5,6,32,37,38], plurality [6,37,41], pre-existing diabetes [35,43,44], essential hypertension [5,6,41], previous SB [5,6,35], sex of baby [35,45], socioeconomic disadvantage [5,6,41,46], accessibility/remoteness [32,46–49], and smoking during pregnancy [5,6, 39,44,50]) and potential factors (health insurance [51] and interpreter utilisation [8]) associated with SB to determine the adjusted OR (aOR). An additional intrapartum-related co-variate, only-midwife accoucheur, was specifically added to the IntraSB analysis. *P* < 0.05 was considered significant, and variables with *p* > 0.1 were removed from the final models [52]. In order to investigate with more depth the relationship between SB and timing of ANC, utilising interpreter services, health insurance status, and type of intrapartum care, the migrant population was further stratified according to the factors of interest as follows: by interpreter utilisation (yes/no) for analysing all SBs combined, by timing of commencement of ANC (first visit before/after week 14) and also by private health insurance status for AnteSB analysis, and by type of intrapartum care (midwife-only/team) for IntraSB analysis in women from African and ‘other’ ethnic backgrounds combined, the only population at-risk of IntraSB. We also limited the intrapartum care analysis to viable births—gestational age at birth >23 completed weeks.

To explore whether the increased risk of AnteSB and/or IntraSB in migrant women are mediated through LBW and PTB, the predicted probabilities of LBW and PTB were estimated from a logistic regression model based on baseline covariates [25,26], including ethnicity, marital status, maternal age group, parity, plurality, presence of medical condition or pregnancy complication, smoking during pregnancy, and socioeconomic disadvantage; then the new binary variables—(Yes/No), ‘high-risk-of-LBW’, and ‘high-risk-of-PTB’—were defined as the predicted probability of LBW and PTB, respectively, above the 95th percentile [26] and were used as additional covariates in the analyses.

#### Sensitivity analyses

Analyses were undertaken by excluding SBs with major anomalies from the analysis because of potential restricted access or differing attitudes to screening or termination of pregnancy reported for some ethnic backgrounds [30,53].

Further, to examine the effect of nonindependence that can arise in the analysis of large population data when women have more than one birth during the period of study, we limited the population of women to those with just one birth record in the data set to examine the effect of nonindependence that can arise in the analysis of large population data. Also, in response to peer review comments, the cluster effect was fitted using the ‘cluster’ option in the STATA package.

Analyses were performed using Stata (version 13·1; StataCorp LP, College Station, Texas).

### Ethics approval

This study was approved by the Human Research Ethics Committee of the WA Department of Health (2015/23). Because of the use of nonidentifiable routinely collected linked administrative health data for the whole population, written consent was not required to conduct the study.

## Results

From 261,430 live and SBs to WA non-Indigenous women during 2005 to 2013, 433 were identified as termination of pregnancy and were excluded. Among the 260,997 births included in the study, 99.0% were delivered in a hospital (258,296), 66.1% to Australian-born women and 33.9% to migrant women. Nonwhite migrants predominantly utilised tertiary and public hospitals, whereas white migrants and Australian-born women had more private hospital separations (Table 1).

**Table 1:** Demographic characteristics of the study population.

Characteristics	Australian-born women	Migrant women							All women	*P*
		White	Asian	Indian	African	Māori	Other	All migrants		
**Total number**	172,571	48,546	18,212	5,503	4,155	2,941	9,038	88,395	260,997	
**Marital status**										<0.001
Never married	18,016 (10.4%)	3,026 (6.2%)	701 (3.9%)	99 (1.8%)	543 (13.1%)	570 (19.4%)	611 (6.8%)	5,550 (6.3%)	23,568 (9.0%)	
Divorced/separated	1,554 (0.9%)	360 (0.7%)	160 (0.9%)	17 (0.3%)	109 (2.6%)	33 (1.1%)	132 (1.5%)	811 (0.9%)	2,366 (0.9%)	
Married/de facto	151,831 (88.0%)	44,693 (92.1%)	17,107 (93.9%)	5,327 (96.8%)	3,449 (83.0%)	2,268 (77.1%)	8,214 (90.9%)	81,058 (91.7%)	232,917 (89.2%)	
Other	1,170 (0.7%)	467 (1.0%)	244 (1.3%)	60 (1.1%)	54 (1.3%)	70 (2.4%)	81 (0.9%)	976 (1.1%)	2,146 (0.8%)	
**Parity**										<0.001
Nulliparous	73,456 (42.6%)	21,205 (43.7%)	8,759 (48.1%)	3,204 (58.2%)	1,217 (29.3%)	955 (32.5%)	3,532 (39.1%)	38,872 (44.0%)	112,340 (43.0%)	
Primiparous	60,403 (35.1%)	17,243 (35.5%)	6,485 (35.6%)	1,817 (33.0%)	1,113 (26.8%)	792 (26.9%)	2,695 (29.8%)	30,145 (34.1%)	90,561 (34.7%)	
Multiparous	38,712 (22.5%)	10,098 (20.8%)	2,968 (16.3%)	482 (8.8%)	1,825 (43.9%)	1,194 (40.6%)	2,811 (31.1%)	19,378 (21.9%)	58,096 (22.3%)	
**Maternal age (years)**										<0.001
Mean (SD)	29.5 (5.6)	31.5 (5.3)	31.2 (4.9)	29.5 (4.4)	28.8 (5.7)	26.8 (6.0)	29.9 (5.6)	30.9 (5.4)	30.0 (5.6)	
<20	7,474 (4.3%)	724 (1.5%)	131 (0.7%)	17 (0.3%)	197 (4.7%)	300 (10.2%)	200 (2.2%)	1,569 (1.8%)	9,045 (3.5%)	
20–24	27,516 (16.0%)	4,364 (9.0%)	1,401 (7.7%)	638 (11.6%)	826 (19.9%)	889 (30.2%)	1,477 (16.3%)	9,595 (10.9%)	37,115 (14.2%)	
25–29	49,076 (28.4%)	11,423 (23.5%)	5,208 (28.6%)	2,288 (41.6%)	1,254 (30.2%)	786 (26.7%)	2,631 (29.1%)	23,590 (26.7%)	72,673 (27.8%)	
30–34	54,744 (31.7%)	17,464 (36.0%)	6,937 (38.1%)	1,869 (34.0%)	1,169 (28.1%)	599 (20.4%)	2,714 (30.0%)	30,752 (34.8%)	85,510 (32.8%)	
35–39	28,412 (16.5%)	11,716 (24.1%)	3,713 (20.4%)	581 (10.6%)	586 (14.1%)	290 (9.9%)	1,612 (17.8%)	18,498 (20.9%)	46,914 (18.0%)	
40–44	5,159 (3.0%)	2,703 (5.6%)	785 (4.3%)	103 (1.9%)	112 (2.7%)	77 (2.6%)	389 (4.3%)	4,169 (4.7%)	9,328 (3.6%)	
>44	190 (0.1%)	152 (0.3%)	37 (0.2%)	<10 (0.1%)	11 (0.3%)	0 (0.0%)	15 (0.2%)	222 (0.3%)	412 (0.2%)	
**Maternal height (cm)**										<0.001
Mean (SD)	165.8 (6.7)	165.1 (6.8)	158.6 (6.0)	159.3 (6.0)	164.5 (7.1)	165.8 (6.0)	161.7 (6.8)	163.0 (7.2)	164.9 (7.0)	
**Medical conditions**										<0.001
Pre-existing diabetes mellitus	1,040 (0.6%)	285 (0.6%)	97 (0.5%)	53 (1.0%)	30 (0.7%)	15 (0.5%)	63 (0.7%)	543 (0.6%)	1,583 (0.6%)	
Hypertension	2,134 (1.2%)	579 (1.2%)	109 (1.6%)	28 (0.5%)	33 (0.8%)	31 (1.1%)	70 (0.8%)	850 (1.0%)	2,984 (1.1%)	
**Smoked in pregnancy**	24,097 (14.0%)	4,161 (8.6%)	317 (1.7%)	40 (0.7%)	78 (1.9%)	1,152 (39.2%)	494 (5.5%)	6,242 (7.1%)	30,342 (11.6%)	<0.001
**Private health insurance**	73,774 (43.1%)	19,247 (40.2%)	5,495 (30.2%)	1,379 (25.0%)	380 (9.2%)	153 (5.3%)	1,471 (16.3%)	59,374 (32.1%)	101,902 (39.4%)	<0.001
**Hospital category**										<0.001
Tertiary	26,337 (15.4%)	8,081 (16.9%)	5,425 (29.8%)	1,956 (35.5%)	1,682 (40.7%)	524 (18.1%)	4,271 (47.6%)	2,1796 (24.9%)	48,278 (18.7%)	
Public metropolitan	39,285 (23.0%)	10,681 (22.3%)	6,268 (34.5%)	2,262 (41.1%)	2,028 (49.0%)	1,424 (49.1%)	2,660 (29.6%)	25,320 (29.0%)	64,611 (25.0%)	
Rural public/private	33,401 (19.5%)	4,834 (10.1%)	1,286 (7.1%)	224 (4.1%)	177 (4.3%)	718 (24.7%)	675 (7.5%)	7,910 (9.0%)	41,317 (16.0%)	
Private metropolitan	72,061 (42.1%)	24,326 (50.8%)	5,201 (28.6%)	1,067 (19.4%)	251 (6.1%)	237 (8.2%)	1,367 (15.2%)	32,446 (37.1%)	104,513 (40.4%)	
**Interpreter service utilised**	19 (0.0%)	306 (0.6%)	1,779 (9.8%)	232 (4.2%)	651 (15.7%)	0 (0.0%)	931 (10.4%)	3,896 (4.5%)	3,918 (1.5%)	<0.001
**Socioeconomic disadvantage**										<0.001
Most disadvantaged	40,521 (23.5%)	6,547 (13.5%)	2,181 (12.0%)	663 (12.1%)	513 (12.4%)	800 (27.2%)	1,276 (14.1%)	11,980 (13.6%)	52,504 (20.1%)	
Remaining population	124,940 (73.0%)	40,013 (83.5%)	15,433 (84.9%)	4,652 (84.4%)	3,496 (84.5%)	2,035 (70.1%)	7,436 (82.9%)	73,053 (83.4%)	198,013 (76.5%)	
**Accessibility/Remoteness Index of Australia**										<0.001
Highly accessible	124,890 (72.4%)	40,836 (84.1%)	16,160 (88.7%)	5,035 (91.5%)	3,830 (92.2%)	2,060 (70.0%)	7,948 (87.9%)	75,869 (85.8%)	200,778 (76.9%)	
Accessible	15,736 (9.1%)	2,390 (4.9%)	474 (2.6%)	78 (1.4%)	51 (1.2%)	224 (7.6%)	205 (2.3%)	3,422 (3.9%)	19,162 (7.3%)	
Moderately accessible	16,125 (9.34%)	2,162 (4.5%)	514 (2.8%)	102 (1.9%)	85 (2.1%)	414 (14.1%)	293 (3.2%)	3,570 (4.0%)	19,699 (7.6%)	
Remote	7,844 (4.6%)	1,282 (2.6%)	396 (2.2%)	70 (1.3%)	39 (0.9%)	116 (3.9%)	231 (2.6%)	2,134 (2.4%)	9,979 (3.8%)	
Very remote	2,062 (1.2%)	415 (0.9%)	97 (0.5%)	23 (0.4%)	23 (0.6%)	49 (1.7%)	92 (1.1%)	699 (0.8%)	2,761 (1.1%)	

Among migrant populations, women from African backgrounds had the lowest proportion of nulliparous women, the highest proportion of interpreter service use (15·7%) at the hospital and post-term pregnancy (2.1%), and resided in very accessible areas in WA (92·2%). Māori women had the highest proportion of socioeconomic disadvantage (27·2%), never married (19.4%), and smoking in pregnancy (39.2%). Migrant women from Indian background had the highest proportion of nulliparous women (58.2%) and experienced the highest prevalence of complications of pregnancy (39.3%), complication of labour (73·0%), and emergency caesarean section (24·4%) (Table 2). Only 5.3% of Māori and 9.2% of African migrants had private health insurance in contrast to 43.1% of Australian-born women. Among Australian-born women, 14% had smoked in pregnancy while only 0.7% and 1.9% of migrants from Indian and African backgrounds, respectively, had smoked in pregnancy.

**Table 2:** Obstetric characteristics of the study population.

Characteristics	Australian-born women	Migrant women							All women	P
		White	Asian	Indian	African	Māori	Other	All migrants		
**All births**	172,571 (66.1%)	48,546 (18.6%)	18,212 (7.0%)	5,503 (2.1%)	4,155 (1.6%)	2,941 (1.1%)	9,038 (3.5%)	88,395 (33.9%)	260,997 (100%)	
**First ANC visit**^1^										
**Median (IQRs)**	10 (8)	12 (10)	11 (12)	11 (13)	15 (15)	14 (16)	15 (14)	12 (12)	11 (10)	
**Mean (SD)**	12.6 (7.4)	13.5 (7.5)	13.8 (8.0)	13.4 (7.9)	16.2 (8.9)	16.4 (9.7)	15.9 (8.8)	14.0 (8.0)	13.1 (7.7)	
**Plurality**										0.025
Singleton	167,481 (97.1%)	47,075 (97.0%)	17,822 (97.9%)	5,389 (97.9%)	4,031 (97.0%)	2,883 (98.0%)	8,725 (96.5%)	85,925 (97.2%)	253,435 (97.1%)	
Multiple	5,090 (2.9%)	1,471 (3.0%)	390 (2.1%)	114 (2.1%)	124 (3.0%)	58 (2.0%)	313 (3.5%)	2,470 (2.8%)	7,562 (2.9%)	
**Pregnancy complications**										<0.001
Gestational diabetes	7,710 (4.5%)	2,732 (5.6%)	2,306 (12.7%)	868 (15.8%)	312 (7.5%)	117 (4.0%)	862 (9.5%)	7,197 (8.1%)	14,907 (5.7%)	
Pre-eclampsia	5,114 (3.0%)	1,194 (2.5%)	325 (1.8%)	124 (2.3%)	127 (3.1%)	77 (2.6%)	216 (2.4%)	2,063 (2.3%)	7,177 (2.8%)	
Any complication	57,596 (33.4%)	15,330 (31.6%)	6,489 (35.6%)	2,162 (39.3%)	1,339 (32.2%)	925 (31.5%)	3,214 (35.6%)	29,459 (33.3%)	87,059 (33.4%)	
**Onset of labour**										<0.001
Spontaneous	83,237 (48.3%)	23,800 (49.0%)	10,804 (59.3%)	2,921 (53.1%)	2,585 (62.2%)	1,975 (67.2%)	5,084 (56.3%)	47,169 (53.4%)	130,428 (50.0%)	
Induced	51,443 (29.8%)	13,356 (27.5%)	3,824 (21.0%)	1,527 (27.8%)	1,037 (25.0%)	680 (23.1%)	2,273 (25.2%)	22,697 (25.7%)	74,147 (28.4%)	
Elective Caesarean	37,891 (22.0%)	11,390 (23.5%)	3,584 (19.7%)	1,055 (19.2%)	533 (12.8%)	286 (9.7%)	1,681 (18.6%)	18,529 (21.0%)	56,422 (21.6%)	
**Complication of labour/delivery**	106,318 (61.6%)	30,258 (62.3%)	12,249 (67.3%)	4,016 (73.0%)	2,833 (68.2%)	1,700 (57.8%)	6,301 (69.7%)	57,357 (64.9%)	163,691 (62.7%)	
**Mode of delivery**										<0.001
Spontaneous vaginal	88,492 (51.3%)	23,187 (47.8%)	8,400 (46.1%)	2,058 (37.4%)	2,531 (60.9%)	2,148 (73.0%)	4,719 (52.2%)	43,043 (48.7%)	131,558 (50.4%)	
Instrumental vaginal	24,796 (14.4%)	7,452 (15.4%)	3,307 (18.2%)	1,252 (22.8%)	386 (9.3%)	237 (8.1%)	1,225 (13.6%)	13,859 (15.7%)	38,660 (14.8%)	
Caesarean	59,283 (34.4%)	17,907 (36.9%)	6,505 (35.7%)	2,193 (39.9%)	1,238 (29.8%)	556 (18.9%)	3,094 (34.2%)	31,493 (35.6%)	90,779 (34.8%)	
**Emergency Caesarean**	26,144 (15.2%)	7,954 (16.4%)	3,484 (19.1%)	1,340 (24.4%)	844 (20.3%)	331 (11.3%)	1,756 (19.4%)	15,709 (17.8%)	41,855 (16.0%)	<0.001
**Accoucheur**										<0.001
Obstetrician	58,780 (34.1%)	16,888 (34.8%)	5,070 (27.8%)	1,131 (20.6%)	370 (8.9%)	254 (8.6%)	1,426 (15.8%)	25,139 (28.4%)	83,926 (32.2%)	
Other medical practitioners	27,837 (16.1%)	9,255 (19.1%)	3,342 (18.4%)	1,422 (25.8%)	1,010 (24.3%)	362 (12.3%)	2,011 (22.3%)	17,402 (19.7%)	45,243 (17.3%)	
Midwife	49,508 (28.7%)	12,903 (26.6%)	4,364 (24.0%)	1,081(19.6%)	1,362 (32.8%)	1,448 (49.2%)	2,470 (27.3%)	23,628 (26.7%)	73,153 (28.0%)	
Mix (team)	36,196 (21.0%)	9,429 (19.4%)	5,406 (29.7%)	1,866 (33.9%)	1,399 (33.7%)	864 (29.4%)	3,112 (34.4%)	2,2076 (25.0%)	58,274 (22.3%)	
Self/no one	250 (0.1%)	71 (0.2%)	30 (0.2%)	<10	14 (0.3%)	13 (0.4%)	19 (0.2%)	150 (0.2%)	401 (0.2%)	
**Sex of baby**										0.980
Boy	88,307 (51.2%)	24,720 (50.9%)	9,466 (52.0%)	2,813 (51.1%)	2,131 (51.3%)	1,490 (50.7%)	4,608 (51.0%)	45,228 (51.2%)	133,549 (51.2%)	
**Gestational age (weeks)**										<0.001
20–27	1,025 (0.6%)	261 (0.5%)	121 (0.7%)	45 (0.8%)	55 (1.3%)	25 (0.9%)	94 (1.0%)	601 (0.7%)	1,626 (0.6%)	
28–31	1,288 (0.8%)	328 (0.7%)	123 (0.7%)	47 (0.9%)	41 (1.0%)	19 (0.7%)	72 (0.8%)	630 (0.7%)	1,919 (0.7%)	
32–36	11,893 (6.9%)	3,172 (6.5%)	1,260 (6.9%)	399 (7.3%)	235 (5.7%)	173 (5.9%)	622 (6.9%)	5,861 (6.6%)	17,754 (6.8%)	
37–41	157,498 (91.3%)	44,520 (91.7%)	16,665 (91.5%)	4,997 (90.8%)	3,737 (89.9%)	2,705 (92.0%)	8,187 (90.6%)	80,811 (91.4%)	238,336 (91.3%)	
≥42	867 (0.5%)	265 (0.6%)	43 (0.2%)	15 (0.3%)	87 (2.1%)	19 (0.7%)	63 (0.8%)	492 (0.6%)	1,362 (0.5%)	
**Birth weight Mean (SD)**	3,379.03 (585.0)	3,371.9 (572.7)	3,217.3 (549.3)	3,114.7 (563.1)	3,269.9 (630.3)	3,403.8 (621.2)	3,274.3 (620.7)	3,310.3 (582.8)	3,355.8 (585.2)	
**Pregnancy outcomes**										
Live birth	171,759 (99.5%)	48,315 (99.5%)	18,117 (99.5%)	5,464 (99.3%)	4,104 (98.8%)	2,923 (99.4%)	8,972 (99.3%)	8,7895 (99.4%)	259,684 (99.5%)	0.001
SB (total)	812 (0.5%)	231 (0.5%)	95 (0.5%)	39 (0.7%)	51 (1.2%)	18 (0.6%)	66 (0.7%)	500 (0.6%)	1,313 (0.5%)	0.001
AnteSB	605 (0.4%)	162 (0.3%)	69 (0.4%)	31 (0.6%)	31 (0.8%)	15 (0.5%)	47 (0.5%)	355 (0.2%)	960 (0.4%)	0.002
IntraSB	185 (0.1%)	57 (0.1%)	24 (0.1%)	<10 (<0.1%)	20 (0.5%)	<10 (<0.1%)	19 (0.2%)	131 (0.2%)	317 (0.1%)	0.001

^1^Gestational age at first ANC visit. Available from January 2010 onwards for 123,655 births (47.4% of the total population).

Note: cells may not add up to 100% because of some variables having multiple values or some characteristics presented in more than one variable.

**Abbreviations:** ANC, antenatal care; AnteSB, antepartum stillbirth; IntraSB, intrapartum still birth; IQR, interquartile range; SB, stillbirth

### AnteSB

In the multivariable analysis, primiparity, living in remote areas, private health insurance, and utilising interpreter services were associated with lower risk of AnteSB (Table 3), whereas age >35 years, multiple pregnancy, pre-existing diabetes, and smoking were associated with increased risk of AnteSB. However, controlling for these factors did not attenuate the odds of AnteSB in African, Indian, and ‘other’ migrant populations; if anything, the OR increased after adjusting for these covariates (Table 3).

**Table 3:** Logistic regression model and the factors associated with AnteSB (2005–2013).

Variables	*N*	Rates^1^	OR (Univariable)	95% CI	aOR (Multivariable)	95% CI
**Migrant status and ethnicity**						
Australian-born (Reference)	605	35	1.00		1.00	
Overseas-born	355	40				
White	162	33	0.95	0.80–1.13	0.94	0.78–1.12
Asian	69	38	1.08	0.84–1.39	1.21	0.93–1.56
Indian	31	56	**1.61**	1.12–2.31	**1.64**	1.13–2.44
African	31	75	**2.14**	1.49–2.07	**2.22**	1.48–2.13
Māori	15	51	1.46	0.87–2.44	1.24	0.74–2.08
Other	47	52	**1.49**	1.10–2.00	**1.46**	1.07–1.97
**Previous SB**			**3.06**	2.18–4.29	**2.67**	1.87–3.81
**Maternal age group**						
20–24 (Reference)					1.00	
<20			1.03	0.73–1.47	0.90	0.62–1.29
25–29			**0.73**	0.60–0.90	**0.81**	0.65–1.00
30–34			0.82	0.67–1.00	1.02	0.83–1.27
35–39			1.01	0.82–1.25	1.22	0.97–1.53
40–44			1.26	0.91–1.74	**1.45**	1.05–2.07
≥45			1.75	0.56–5.51	1.20	0.30–4.90
**Parity**						
Nulliparous					1.00	
Primiparous			**0.78**	0.68–0.90	**0.76**	0.65–0.88
Multiparous			1.10	0.94–1.29	0.89	0.75–1.06
**Plurality**						
Singleton pregnancy					1.00	
Multiple pregnancy			**3.98**	3.23–4.90	**3.99**	3.24–4.95
**Medical conditions/pregnancy complications**						
Pre-existing diabetes mellitus			**2.08**	1.18–3.69	**1.90**	1.07–3.38
Essential hypertension			**1.66**	1.04–2.64	1.53	0.95–2.48
**Smoked in pregnancy**			**1.40**	1.17–1.68	**1.30**	1.07–1.56
**Socioeconomically disadvantaged**^2^			**1.18**	1.01–1.37	1.16	0.98–1.38
**Accessibility/Remoteness Index of Australia**						
Highly accessible			1.00		1.00	
Accessible			1.07	0.85–1.36	1.06	0.83–1.35
Moderately accessible			1.17	0.93–1.46	1.06	0.82–1.36
Remote			0.68	0.45–1.00	**0.65**	0.44–0.98
Very remote			0.88	0.46–1.70	0.90	0.46–1.74
**Private health insurance**			**0.64**	0.56–0.74	**0.68**	0.58–0.79
**Interpreter service utilised**			**0.43**	0.21–0.92	**0.33**	0.16–0.71

^1^Cumulative incidence rates are per 10,000 total births.

^2^The bottom 20% of Index of Relative Socioeconomic Disadvantage was compared with the remaining population.

Bolded values are *P* < 0.05.

Note: Factors in the table are those that have been included in the multivariable (adjusted model) analysis. These factors are not mutually exclusive and might coincide in the same woman.

**Abbreviations:** AnteSB, antepartum stillbirth; aOR, adjusted odds ratio; OR, odds ratio; SB, stillbirth

### IntraSB

Female offspring, parity, and socioeconomic disadvantage were associated with lower risk of IntraSB. Conversely, multiple pregnancy, pre-existing diabetes, age >35 years, living in very remote areas, smoking, and midwife-only accoucheur were associated with higher risk of IntraSB; however, controlling for these factors did not explain and even increased the effect measure for IntraSB in women from African and ‘other’ ethnic backgrounds (Table 4).

**Table 4:** Logistic regression model and the factors associated with IntraSB (2005–2013).

Variables	*N*	Rates^1^	OR (Univariable)	95% CI	aOR (Multivariable)	95% CI
**Migrant status and ethnicity**						
Australian-born (reference)	185	11	1.00		1.00	
Overseas-born	131	15				
White	57	12	1.10	0.81–1.47	1.00	0.74–1.36
Asian	24	13	1.23	0.80–1.88	1.36	0.93–2.24
Indian	<10	15	1.36	0.67–2.75	1.60	0.78–3.30
African	**20**	**48**	**4.51**	2.84–7.16	**5.24**	3.35–8.91
Māori	<10	10	0.95	0.30–2.98	0.75	0.24–2.36
Other	**19**	**21**	**1.96**	1.22–3.15	**2.18**	1.34–3.54
**Previous SB**			**4.32**	2.61–7.15	**6.51**	3.79–11.20
**Year of birth**						
2005 (reference)						
2006			1.58	0.93–2.68	1.62	0.96–2.76
2007			1.63	0.97–2.74	**1.74**	1.03–2.94
2008			1.31	0.76–2.25	1.34	0.78–2.30
2009			1.37	0.80–2.33	1.44	0.84–2.46
2010			1.09	0.62–1.91	1.22	0.70–2.14
2011			1.36	0.80–2.31	1.44	0.85–2.46
2012			1.64	0.99–2.72	**1.69**	1.01–2.82
2013			1.19	0.70–2.04	1.22	0.71–2.10
**Baby sex**						
Female			**0.77**	0.61–0.96	**0.74**	0.59–0.93
**Maternal age group**						
20–24 (reference)					1.00	
Less than 20			1.09	0.59–2.01	0.85	0.44–1.67
25–29			0.84	0.59–1.20	1.10	0.79–1.66
30–34			0.85	0.60–1.20	1.37	0.96–2.04
35–39			0.98	0.68–1.43	**1.72**	1.18–2.70
40–44			1.30	0.74–2.29	**2.44**	1.43–4.83
≥45			1.84	0.25–13.36	2.34	0.36–17.99
**Parity**						
Nulliparous (reference)					1.00	
Primiparous			0.81	0.63–1.04	**0.56**	0.44–0.73
Multiparous			**0.69**	0.50–0.93	**0.31**	0.23–0.50
**Plurality**						
Singleton pregnancy (reference)					1.00	
Multiple pregnancy			**8.04**	6.08–10.64	**16.42**	12.03–22.40
**Medical conditions/Pregnancy complications**						
Pre-existing Diabetes Mellitus			2.24	0.83–6.00	**3.13**	1.15–8.55
**Smoked in pregnancy**			**1.45**	1.07–1.96	**1.53**	1.10–2.09
**Socioeconomically disadvantaged**^2^			**0.68**	0.50–0.93	**0.67**	0.48–0.94
**Accessibility/Remoteness Index of Australia**						
Highly accessible (reference)					1.00	
Accessible			0.69	0.42–1.13	0.82	0.50–1.33
Moderately accessible			0.63	0.38–1.05	0.71	0.41–1.20
Remote			0.86	0.47–1.57	0.84	0.44–1.59
Very remote			**2.27**	1.12–4.59	**2.95**	1.44–6.07
**Birth attendant/supervisor**^3^						
Only midwife			**5.05**	4.00–6.38	**9.00**	6.85–11.82

^1^Cumulative incidence rates are per 10,000 total births.

^2^The bottom 20% of Index of Relative Socioeconomic Socioeconomic Disadvantage was compared to the remaining population.

^3^Compared to all other care in labour combined.

Bolded values indicate *P* < 0.05.

Note: Factors in the table are those that have been included in the multivariable (adjusted model) analysis. These factors are not mutually exclusive and might coincide in the same woman.

**Abbreviations:** aOR, adjusted odds ratio; IntraSB, intrapartum stillbirth; OR, odds ratio; SB, stillbirth

When the analysis was undertake on viable birth (>23 weeks gestation), the higher odds of IntraSB remained significant in women from African background (aOR 4.78, 95% CI 1.86–12.30) but lost significance in women from ‘other’ ethnic backgrounds (aOR 1.96, 95% CI 0.78–4.97).

### ANC

**Fig 2:**
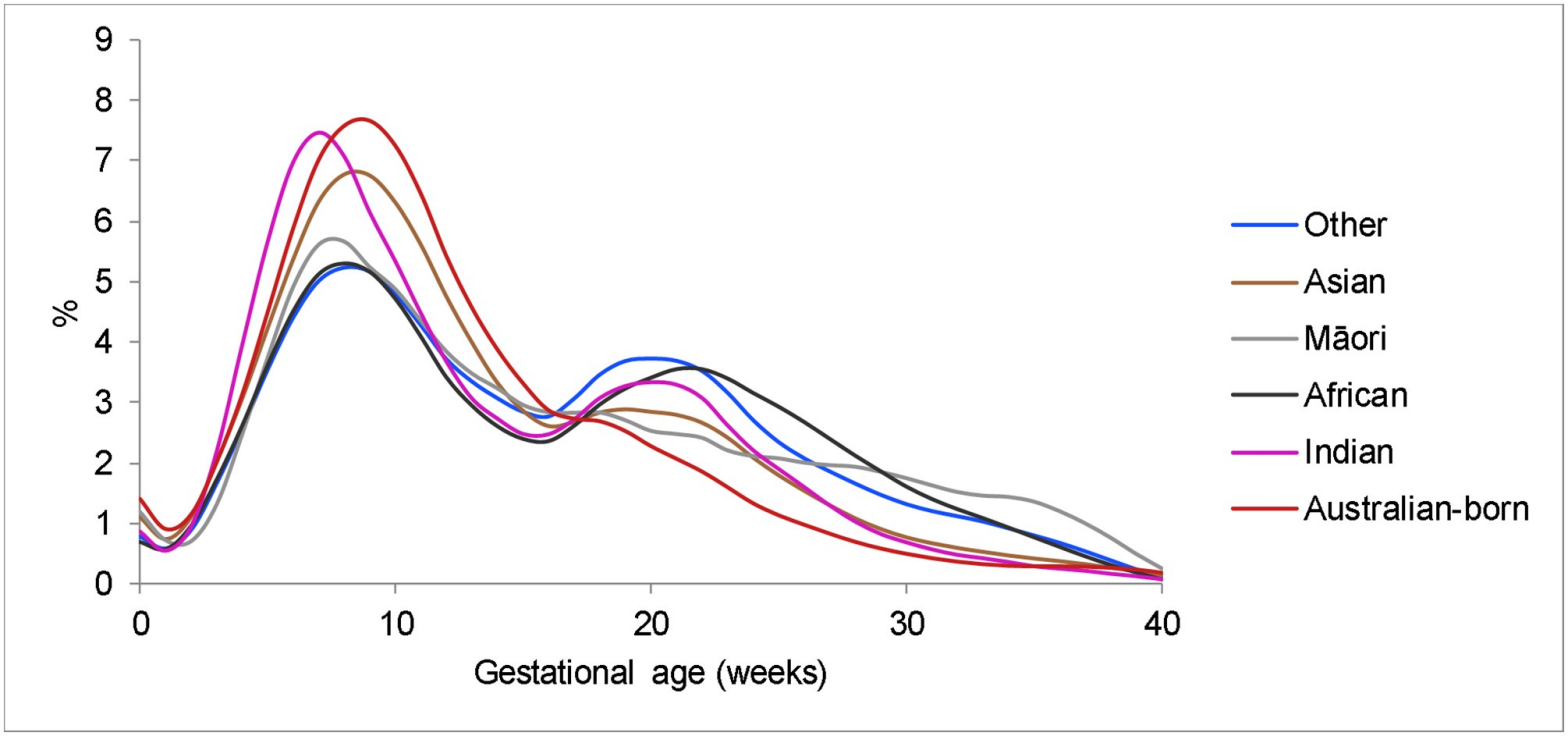
Distribution of gestational age at first ANC visit for specified study groups (2010–2013). ANC, antenatal care.

Migrant women commenced ANC visits (ANC visit) 1.5 weeks later (*P* < 0.001) than Australian-born women (Table 2). African and ‘other’ migrant women, specifically, commenced ANC 5 weeks later than Australian-born women (*P* < 0.001): more than 50% had their first visit after week 14 of pregnancy (Table 2 and Fig 2).

When the population was stratified by late ANC booking, the increased risk of AnteSB was confined to those who booked late in Indian (OR 1.49, 95% CI 0.85–2.60 versus Late OR 2.16, 95% CI 1.18–3.95), Māori (OR 0.90, 95% CI 0.29–2.81 versus OR 3.03, 95% CI 1.43–6.44) and ‘other’ migrant women (OR 1.08, 95% CI 0.57–2.04 versus OR 2.19, 1.34–3.58). This finding was not observed in migrant women from African backgrounds and remained the same in the adjusted analysis (Table 5).

**Table 5:** Comparison of the odds of AnteSB in migrant women, stratified by ethnicity and timing of first ANC visit, with Australian-born women (2010–2013).

Characteristics	AnteSB		
Australian-born (Ref)^1^ (*N* = 76,875)	1.00		
	First ANC visit in pregnancy		
Migrant women (*N*)	At any time (all) aOR (95% CI)^2^	At/before week 14 aOR (95% CI)^2^	After week 14 aOR (95% CI)^2^
White (23,162)	1.06 (0.83–1.36)	1.05 (0.82–1.35)	0.89 (0.58–1.37)
Asian (10,514)	1.30 (0.93–1.83)	1.41 (0.96–2.10)	1.05 (0.58–1.91)
Indian (4,069)	**1.91 (1.25–2.94)**	1.66 (0.94–2.92)	**2.27 (1.23–4.21)**
African (2,303)	**1.95 (1.16–3.26)**	**2.52 (1.36–4.67)**	1.29 (0.56–2.16)
Māori (1,681)	1.42 (0.75–2.69)	0.73 (0.23–2.30)	**2.33 (1.09–5.01)**
Other (5,047)	**1.53 (1.02–2.29)**	1.03 (0.52–1.92)	**2.09 (1.26–3.46)**

^1^The reference group was comprised of all Australian-born women regardless of ethnicity and timing of first ANC visit.

^2^Adjusted for previous stillbirth, maternal age group, parity, plurality, socioeconomic status, remoteness/accessibility, pre-existing diabetes mellitus, smoking in pregnancy, interpreter use and private health insurance status

Bolded values indicate *P* < 0.05.

**Abbreviations:** aOR, adjusted odds ratio; AnteSB, antepartum stillbirth

### Birth attendant and intrapartum care

**Fig 3:**
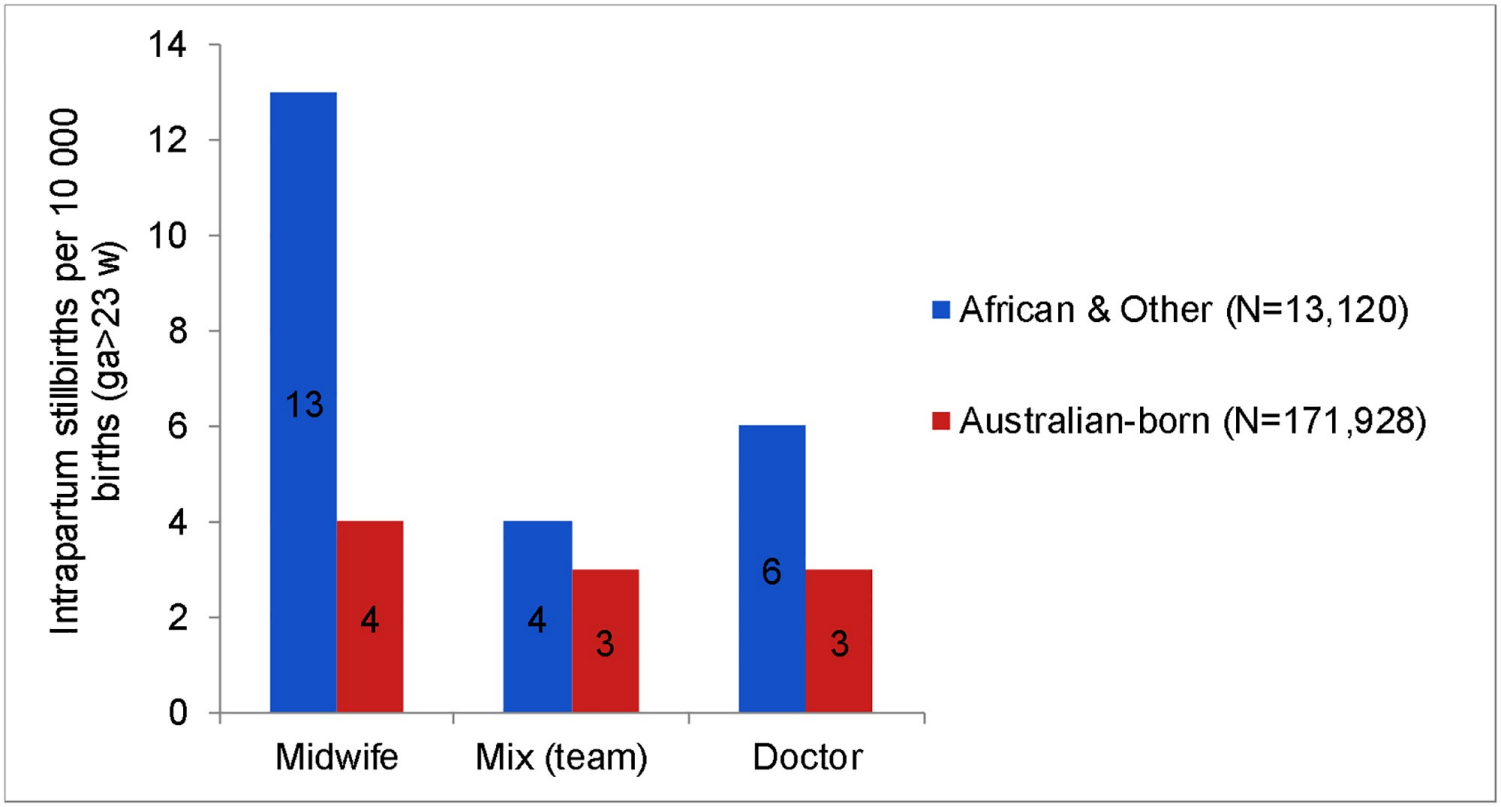
Unadjusted cumulative incidence rate of intraSB (>23 weeks gestation) by intrapartum care provider (2005–2013). Note: Doctor Birth attendant/supervisor indicates that birth was attended by an obstetrician and/or by other medical practitioners, whereas Mix (team) attendant/supervisor refers to occasions when both doctor and midwife were present at birth. intraSB, intrapartum stillbirth.

When the birth attendant was a midwife, with no accompanying doctor, the odds of IntraSB in African and ‘other’ women was more than 3 times that of Australian-born women (OR 3.43, 95% CI 1.28–9.19), which remained significant after adjusting for other factors. However, when the intrapartum care was provided by mix attendants (team), the odds were the same (OR 1.34, 95% CI 0.30–5.98) with no difference after adjustment. For viable births (>23 weeks gestation), intrapartum care with midwife, mix (team), and doctor attendants showed similar IntraSB rates in Australian-born women (Fig 3). In contrast, IntraSB rate was more than 3-fold (*P* = 0.009) higher in African and ‘other’ migrants than Australian-born women with midwife-only attendants. No difference in IntraSB rates of migrant and Australian-born women was observed with intrapartum care by doctor or by mix (team) attendants (Fig 3). No IntraSB was observed in African or ‘other’ women when birth was attended by an obstetrician (*N* = 1,793).

### Interpreter service

**Fig 4:**
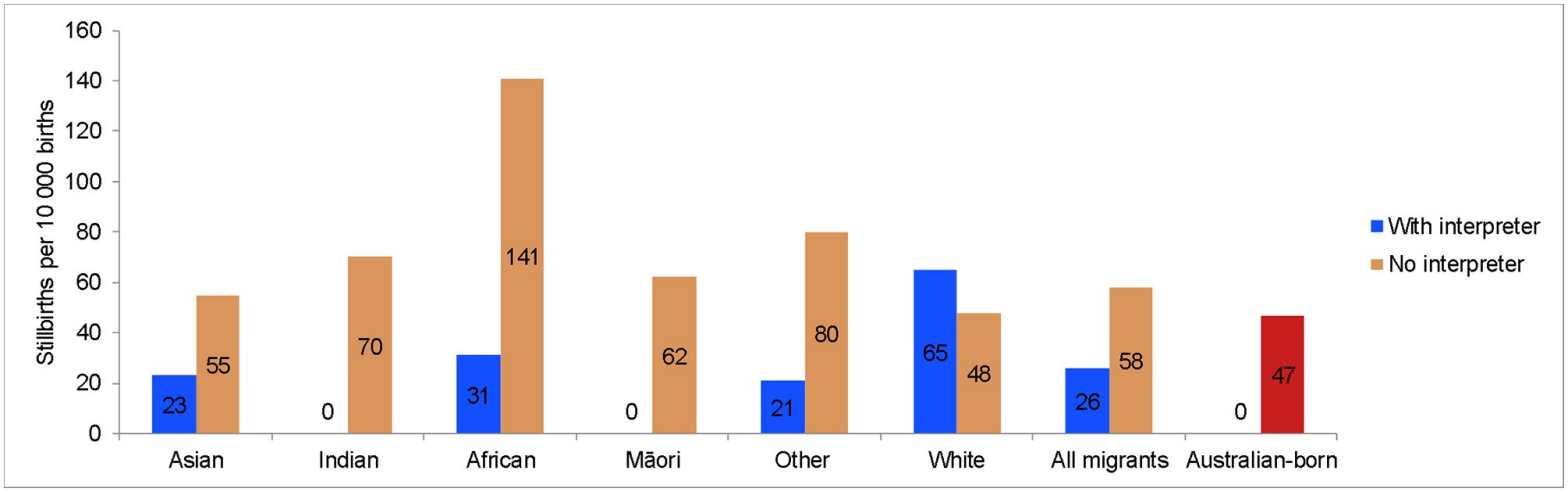
The cumulative incidence rates of overall SB according to interpreter service use (2005–2013). SB, stillbirth.

The rate of overall SB was lower (26 versus 58 per 10,000 births, *P* = 0.011) in migrant women who utilised interpreter services from those who did not, especially among African women (31 versus 141 per 10,000 births, *P* = 0.034; Fig 4). Compared with Australian-born women, migrants who utilised interpreter service had lower odds of SB (OR 0.51; 95% CI 0.27–0.96), whereas their counterparts who did not have an interpreter had higher odds (OR 1.20; 95% CI 1.07–1.35).

### Controlling for the effect of LBW and PTB

High-risk-for-LBW and high-risk-for-PTB were both strongly associated with higher odds of AnteSB (OR 4.47; 95% CI 3.80–5.25, OR 4.13; 95% CI 3.50–4.88, respectively) and IntraSB (OR 7.01; 95% CI 5.46–8.99, OR 6.34; 95% CI 4.92–8.19, respectively). Adjusting for High-risk-for-LBW attenuated all the increased risk of AnteSB for Indian women (aOR 1.16; 95% CI 0.77–1.73) but not for African (aOR 2.02; 95% CI 1.39–2.95) or ‘other’ (aOR 1.41; 95% CI 1.04–1.91) women. Adjusting for High-risk-for-PTB did not alter the odds of AnteSB or IntraSB for any ethnicity.

### Private health insurance

Compared to Australian-born women, women from an Indian background who had private health insurance did not experience increased odds of AnteSB (aOR 1.14, 95% CI 0.47–2.77), whereas those who did not have private health insurance showed higher odds (aOR 2.04, 95% CI 1.35–3.09).

### Sensitivity analysis

Removing SBs with major anomalies or limiting the population of study to women with only one birth record in the data set did not result in appreciable difference in primary findings.

## Discussion

In this linked data study, we showed that having private health insurance and utilising interpreter services were associated with a lower risk of AnteSB and that midwife-only accoucheur was associated with a higher risk of IntraSB. Yet, controlling for these factors, in addition to other factors, did not completely explain the increased risk of either AnteSB in Indian, African, and ‘other’ ethnic migrants or IntraSB in African and ‘other’ migrants. In further stratified analyses, late commencement of ANC visit and lack of access to or uptake of intrapartum care by doctors emerged as underlying factors for the increased risk of SB in at-risk groups of migrants. Migrant women from an Indian background who had private health insurance did not experience increased odds of AnteSB, and those from African and ‘other’ backgrounds who had a doctor-midwife team intrapartum care did not have a higher rate of IntraSB compared with Australian-born women.

Indian migrants, as a population, commenced ANC visits early, and 25% had private health insurance, yet they had a high rate of AnteSB. Stratifying the population by the timing of ANC visit in the analysis confirmed that the increased AnteSB was unique to Indian women who booked late. Being high-risk-for-LBW regardless of cause, PTB or fetal growth restriction (FGR) [41,44], had a great impact on AnteSB in Indian migrants. This suggests that early engagement with the healthcare system may reduce the number of SBs, perhaps through implementing interventions that prevent PTB and/or FGR, particularly in this group. Contrary to the reports from the United States [54], having private health insurance was associated with a reduced risk of SB in this group in our study. Perinatal mortality disparities between public and private care, though not stratified by ethnicity, have been reported in Queensland, Australia, as well [51].

Detecting third-trimester FGR is challenging; clinical assessment misses approximately one-third of cases [55], ultrasound assessment is costly, and the most appropriate ultrasound biometry charts to use, especially for nonwhite ethnic minorities and naturally short-stature, are controversial [56–58]. It is of note that migrant women of Asian ethnicity, despite having a similar height to Indian women, did not have a high rate of AnteSB in our study. However, a larger proportion of them had private health insurance, an obstetrician accoucheur, and delivered at a private hospital, which may indicate better access to ultrasound during pregnancy. The finding that controlling for high-risk-for-LBW status attenuated all the increased odds of AnteSB in women from Indian background may also indicate the difficulty with detecting FGR, especially in those who commence ANC visits late. Universal third-trimester ultrasonography in the United Kingdom tripled detection of small for gestational age infants at risk of adverse perinatal outcomes [59]. Thus, improving access to more frequent ultrasound surveillance during pregnancy and third-trimester for migrant women of Indian ethnicity in public settings may afford a simple intervention to reduce the rate of AnteSB in this high-risk group.

Stratifying by the timing of the first ANC visit also showed that in women from ‘other’ backgrounds the odds of AnteSB were exclusively increased in those who commenced ANC later than 14 weeks. Further, by removing previable births, the increased odds of IntraSB in this group attenuated. Timing of first ANC visit is important for ensuring optimal pregnancy outcomes and late booking may result in loss of opportunity for potential comorbidity diagnosis and intervention [22,60]. Moreover, interventions with demonstrated success in preventing PTB in some women (such as aspirin, progesterone, and cervical cerclage) are only effective when started early in pregnancy, especially for previable PTB prevention [61,62]. Interventions can only be offered if women engage early with ANC.

Migrant women who utilised interpreter services had lower odds of SB than those who did not, particularly in women of African ethnicity. Interpreter use among Chinese-born women residing in WA has been shown to be associated with lower rates of PTB and close to the rate of PTB reported in China [63]; to our knowledge, this is the first time that this factor has been examined in relation to the risk of SB. We were surprised by the lower rates of SB in migrants who utilised an interpreter as the majority of nonwhite migrants in WA are from regions with high rates of SB [6]. Variation in rates of interpreter use between migrant groups may reflect differences in English comprehension, willingness to utilise interpreters, and/or culture; evidence shows that in some ethnic groups, the husband acts as the interpreter or insists to provide language support for women even when accredited interpreters are available [8]. The relationship between no interpreter service utilised and higher rates of SB is concerning; it is not clear whether this group had enough English language proficiency or did not use interpreter because of lack of/reluctance to access such services. The finding that migrant women who used an interpreter had a lower rate of SB may indicate the provision of a more culturally responsive healthcare service; thus, this may afford an opportunity for intervention to reduce the rate of SB.

Experts have previously expressed concern over the lack of access to interpreter services, often because of unfamiliarity of migrants with the Australian health system and lack of awareness of their entitlement to nation-wide interpreter services free of charge [64–66]. The Doctors Priority Line, run through the Department of Immigration and Citizenship’s Translating and Interpreting Service, links interpreters in over 160 languages, 24 hours per day, 7 days per week to doctors by telephone within 3 minutes of their call and can arrange on-site interpreters as well [66]. Yet, use of family members instead of professional interpreters was reported in at least 49% of patients during their inpatient stay in a study [65]. Although it is well established that language barriers are linked to lower quality of care, misconceptions about the use of an interpreter being costly, time-consuming, or threatening confidentiality, or even uncertainty about the responsibility for contacting interpreter services by healthcare providers may also play a role in low uptake of this national fee-free service that is unique in the Anglophone world [66–68].

In this study, midwife-only accoucheur was associated with increased IntraSB even after removing previable births. To date, this area has not been thoroughly evaluated because of the large sample size required for adequate statistical power. In New Zealand, where the majority of births are delivered by midwives, an unexplained excess in adverse birth outcomes with midwife-led births compared with medical-led births was reported [69]. Despite 244,047 pregnancies, that study was underpowered to evaluate SB (321 SBs) and could not differentiate the type of SB [69] Further, the study could not control for confounders such as place of residence [70]. We had a substantially larger sample size (1,313 SBs), adjusted for accessibility/remoteness and socioeconomic disadvantage and knew the type of SB; midwife-only intrapartum care was still associated with increased risk of IntraSB after 23-weeks’ gestation in migrants but not in the Australian-born population. The exact mechanism for this is unclear and warrants further research as IntraSB rates in African and ‘other’ migrants with doctor-midwife (team) intrapartum care were similar to Australian-born women. Of note, the African women had also 4-fold higher rates of post-term pregnancy compared with Australian-born women as well. Whether this indicates a ‘preference’ for a model of care for African and other ethnic women in choosing midwife-only care, reluctance to undergo medical interventions (e.g., instrumental/caesarean delivery) [71,72] or lack of access to doctors because of logistics or timing of reaching facilities is not understood. Future investigation is required to understand this difference and to develop strategies to reduce IntraSB in this at-risk migrant group.

### Other findings

SB rates have previously been reported to be higher in remote and very remote areas in Australia [48]; in this study, we found the rate of AnteSB in remote areas was decreased. This observation may be because of previous studies reporting all SBs, whereas we have dichotomised into AnteSB and IntraSB. Further, in WA, high-risk pregnancies are typically transferred to regional centres or tertiary hospitals in the state capital; hence the population remaining in remote and very remote areas are, by design, at low risk of AnteSB [49,73,74]. In contrast, the increased rate of IntraSB in the very remote area may indicate a lack of timely access to emergency intervention, because of late arrival at the facility or long decision delivery interval in obstetric emergencies for which we did not have data to examine [75,76]. Additional information about the WA healthcare system and the current model of care to improve rural perinatal outcomes is provided in the S1 WA Health. It should be noted, however, that the majority of migrant women lived in highly accessible areas and major cities in our population. Thus these findings cannot be used to draw inferences for specific ethnic groups’ risk of SB in relation to remoteness because of very small numbers. Thus, the observed decreased and increased risk of AnteSB and IntraSB in remote and very remote regions, respectively, are effectively showing such risk in the non-Indigenous Australian-born populations living in those regions relative to those living in highly accessible regions.

The socioeconomic disadvantage being protective of IntraSB was an unexpected finding and may be because of factors unable to adjust for such as (lower) body mass index (BMI) [77] or healthcare options available to these women such as doctor-midwife (team) care. It was noted that 23.5% of Australian-born versus 13.5% of the migrant population was in this socioeconomic disadvantage category.

### Generalizability and clinical relevance

The reported findings can be generalized to high-income settings serving migrant residents from similar ethnic backgrounds, including other states of Australia, Europe, and Canada. These findings are of particular relevance to clinical practice. Primary healthcare services can be utilised to improve health literacy and familiarity with the health system for migrant women. This can include information on the necessity of early engagement with ANC program, the crucial role of communication and effective use of interpreter services, and the value of doctor-midwife (team) intrapartum care. Developing tools and guidelines for healthcare providers to assess their patient’s sufficient ability to communicate in English is crucial to ensure mutual understanding and effective transfer of information.

### Strength

This is by far the most comprehensive SB study on pregnancy outcomes of migrants from diverse ethnic backgrounds in Australia. Access to a variety of databases and numerous variables made cross-source ascertainment of exposure and outcomes possible and optimized accuracy and completeness of data. Capability to differentiate the type of SB for 99.98% SBs to investigate intrapartum factors, not previously undertaken, can guide appropriate policy and practice to reduce IntraSBs in migrants and in very remote areas.

### Limitations

The main limitation of this study was that gestational age at first ANC visit was recorded from 2010 onwards and was only available for 4 years of our study period. Thus, the related analyses reported were only possible for almost a half of the whole study population.

We used whole population linked data for this study. One limitation with analysis of large population data sets can be the nonindependence of data arising from the occasions women have had more than one birth or SB during the study period. In this study, however, accounting for clustering or limiting the analyses to women with only one birth record, did not affect the results.

Another limitation is the risk of misclassification because of the use of linked administrative health data that have not been collected for the purpose of this specific research. We conducted cross-source ascertainment through multiple data sets we had access to in our study; however, a residual risk of misclassification towards the null for the analyses used ethnicity (only available from MNS) may still remain. For example, Indian women with a high risk of AnteSB have been misclassified as Asian. In such a case, the actual risk of AnteSB can be higher than reported. However, such risk is very low as a validation study of MNS has confirmed the reliability of this database with a Proportion Records Correct of 94.1% for the variable ethnicity [78].

Also, the standard of care may change over time and create bias. We have adjusted the analysis for the year of birth to avoid such bias in our study.

Moreover, confounding because of covariates not available in the data set (i.e., BMI given that obesity is associated with increased risk of SB) may also be present [44,77]. However, we have adjusted the analyses for many covariates including pre-existing diabetes mellitus and essential hypertension that are associated with high BMI.

Further, in the LBW/PTB analysis, the predictive power of LBW/PTB depends on the size of the baseline covariates. Thus, a larger set of variables may change the estimates obtained to designate ‘high-risk’ status [26].

Also, ARIA may not show intracity difficulties individuals may face for accessing services. The accoucheur variable only records the birth attendant; neither the ANC provider nor the time from seeking intrapartum care to delivery was known.

Finally, despite the use of a large whole population data set, it should be noted in the interpretation of findings, multiple comparisons such as those in the adjusted intrapartum-related analyses may have led to small numbers of samples in those analyses.

Considering all the above, the results should be interpreted with caution.

### Conclusion

This retrospective cohort study showed that the pattern of healthcare and service utilisation in pregnant migrant women differs from that of Australian-born women and may be contributing to the increased risk of SBs in African, Indian, Māori, and ‘other’ ethnic populations in WA. Thus, to reduce SB rates in the migrant population, modifying both women’s attitudes towards the health system and also certain aspects of health services are required.

Raising awareness of the importance of proactively seeking ANC and using/offering interpreter services for migrant women of reproductive age is vital. For healthcare providers and policymakers, this strategy has the potential to be used as an intervention to reduce the risk of SB. Culture-oriented educational programmes/campaigns may also help to address the concerns of this at-risk group and facilitate greater engagement with the healthcare system early in pregnancy. Improving access to doctor-midwife (team) intrapartum care for African and ‘other’ migrant populations, as well as the provision of routine third-trimester ultrasound surveillance for migrant women from an Indian background, to monitor fetal growth, may also reduce the rates of SB.

Given these findings highlight the influence of service utilisation on the risk of SB, investigation of the acculturation-related factors on the risk of SB, especially the length of residence in Australia, age on arrival, and intermarriage that can give an indication of familiarity with the health system and also competency in English language and communication, in migrant women is warranted.

## Supporting information

S1 WA Health Overview of the WA health system. WA, Western Australia.
(DOCX)

S1 RECORD Checklist (DOCX)

## Abbreviations

ANC: antenatal care
AnteSB: antepartum stillbirth
aOR: adjusted odds ratio
ARIA: Accessibility/Remoteness Index of Australia
BMI: body mass index
FGR: fetal growth restriction
HMDC: Hospital Morbidity Data Collection
IntraSB: intrapartum stillbirth
IRSD: Index of Relative Socioeconomic Disadvantage
LBW: low birth weight
MNS: Midwives Notification System
OR: odds ratio
PTB: preterm birth
SB: stillbirth
WA: Western Australia
WADLS: WA Data Linkage System
WARDA: WA Registry of Developmental Anomalies
