# Supplementary material for: Healthcare factors associated with the risk of antepartum and intrapartum stillbirth in migrants in Western Australia (2005-2013): A retrospective cohort study
Source: PLoS Med. 2020 Mar 17;17(3):e1003061. doi: 10.1371/journal.pmed.1003061 (PMC7077810; doi:10.1371/journal.pmed.1003061)
Supplement: S1 WA Health — WA, Western Australia. (DOCX) [file pmed.1003061.s001.docx]

**The Health System of Western Australia**

Australia’s universal health care system is called Medicare. The Australian government pays for Medicare through the Medicare levy which is paid by working Australians as part of their income tax [1]. Western Australia’s health system (WA Heath) is a mix of services provided by the Australian and State Governments and by private healthcare providers. There are three types of hospitals: public hospitals managed by the WA Health, private hospitals managed by private organisations, and public hospitals run in partnership with private organisations. An individual can be admitted as either a public or private patient to a public hospital. Private patients in public hospitals may choose their treating doctor where possible, while a private patient in a private hospital can choose their doctor, hospital and when to be treated [2].

WA Health has more than 80 hospitals spread across an area of 2.5 million square kilometres, providing service to approximately 2.5 million people. These include 6 tertiary hospitals including the sole tertiary perinatal centre with state-of-the-art medical facilities, 4 general and 2 specialised non-teaching hospitals, 9 large rural and numerous country hospitals, and 3 Private Public Partnership hospitals delivering free public healthcare to the community [3].

Hospital Morbidity Data Collection (HMDC) is one of the largest data collections managed by the WA Health and contains information related to all inpatient discharge summary data from all public and private hospitals in WA [4]. The HMDC is a key information source to meet the mandatory and statutory reporting requirements. It is comprised of more than 22,000,000 electronic inpatient records dating back to 1970 and increases every year in line with population growth [4]. Fig 1 shows the number and the distribution of hospitals across WA [5].


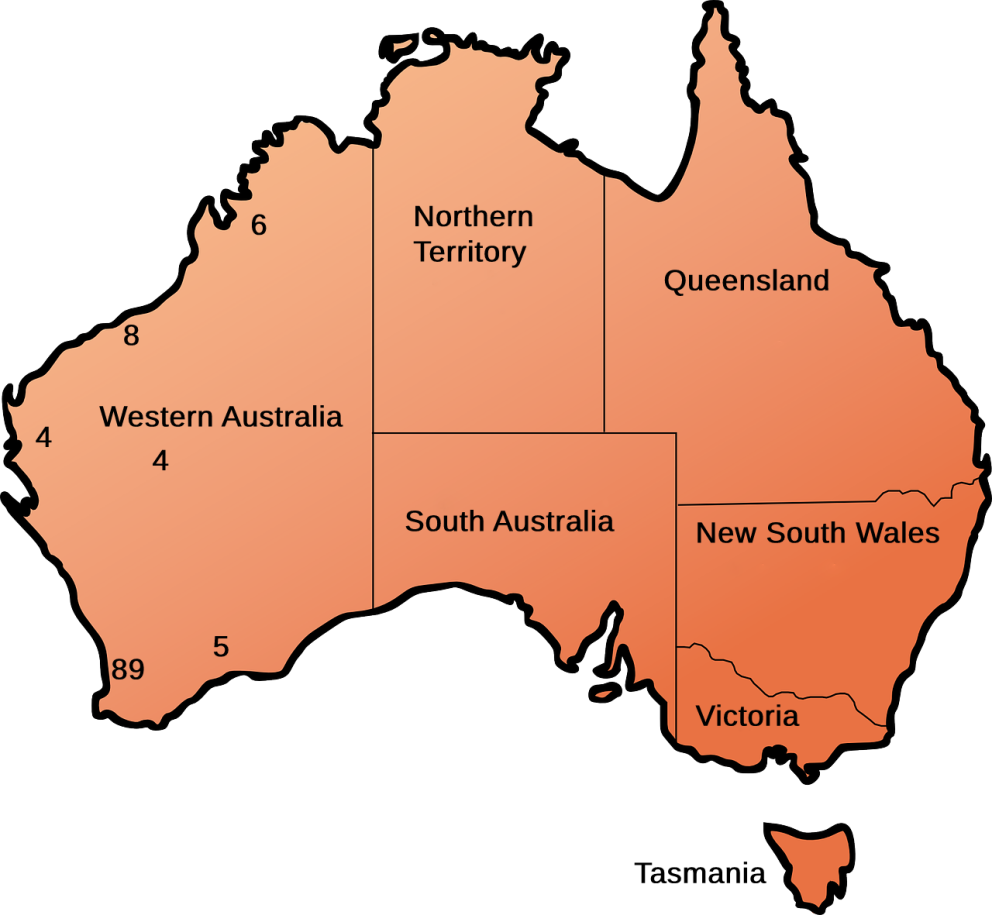


**Fig 1. The distribution of hospitals (public and private) in WA.** Base image by [OpenClipart-Vectors](https://pixabay.com/users/OpenClipart-Vectors-30363/?utm_source=link-attribution&amp;utm_medium=referral&amp;utm_campaign=image&amp;utm_content=146505) from [Pixabay](https://pixabay.com/?utm_source=link-attribution&amp;utm_medium=referral&amp;utm_campaign=image&amp;utm_content=146505).

**Remoteness in Western Australia**

The Australian Statistical Geography Standard defines Remoteness Areas into 5 classes of remoteness: Major Cities of Australia, Inner Regional Australia, Outer Regional Australia, Remote Australia, and Very Remote Australia [6]. This classification is intended to allow users to make comparisons and undertake statistical analysis to inform research and policy development. The Accessibility and Remoteness Index of Australia (ARIA), produced by the Hugo Centre for Migration and Population Research at the University of Adelaide is used to measure access to services [6]. This index is derived by measuring the road distance from a point to the nearest Urban Centres and Localities in five separate population ranges of Highly Accessible, Accessible, Moderately Accessible, Remote, and Very remote. Some of the most remote areas in Australia, albeit with low population density [7], are situated in WA.

**Aeromedical transfer of high-risk pregnancies in WA**

Complicated births to rural mothers are at a higher risk of stillbirth and neonatal mortality than urban infants in Australia [8, 9]. To improve perinatal outcomes of WA rural population, women with high-risk pregnancies, such as those at risk of preterm labour, have been transferred by Royal Flying Doctor Service from rural areas to the sole tertiary perinatal centre, King Edward Memorial Hospital (KEMH), for decades in this state [8, 10, 11]. Fig 2 shows WA geographic regions and the numbers of transfers from each region from September 2007 to 31 December 2009 based on a published article [10]. Please note the distance from Perth in which KEMH is located.


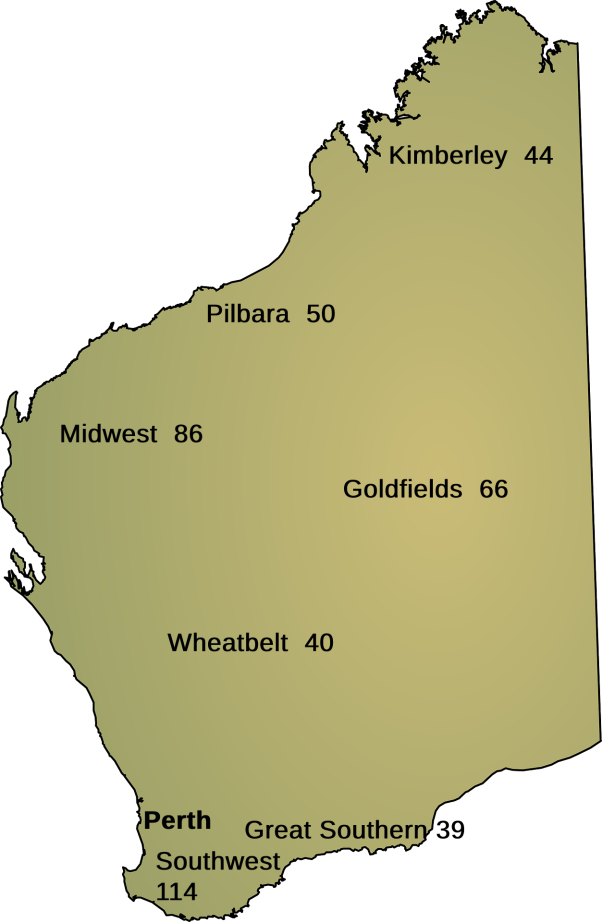


**Fig 2. Number of transfers from each geographic region of Western Australia** **(September 2007 to 31 December 2009).** Base image by [OpenClipart-Vectors](https://pixabay.com/users/OpenClipart-Vectors-30363/?utm_source=link-attribution&amp;utm_medium=referral&amp;utm_campaign=image&amp;utm_content=146505) from [Pixabay](https://pixabay.com/?utm_source=link-attribution&amp;utm_medium=referral&amp;utm_campaign=image&amp;utm_content=146505).

**References**

1. Australian Government Department of Human Services. About Medicare [Cited January 2020]. Available from: <https://www.humanservices.gov.au/individuals/subjects/whats-covered-medicare/about-medicare>
2. Government of Western Australia. Overview of the WA Health System. 2019 [Cited January 2020]. Available from: <https://healthywa.health.wa.gov.au/Articles/N_R/Overview-of-the-WA-health-system>.
3. Government of Western Australia. Department of Health. Hospital Information. 2019 [Cited January 2020]. Available from:

<https://ww2.health.wa.gov.au/About-us/Hospital-Information>.

1. Government of Western Australia. Hospital Morbidity Data System. REFERENCE MANUAL PART A: Contacts, Hospital Responsibilities, Data Element Definitions.V1.0. 20172017 [Cited January 2020]. Available from <https://ww2.health.wa.gov.au/~/media/Files/Corporate/general%20documents/Clinical%20coding/Guides%20and%20summaries/HMDS-REF-Manual-PartA.pdf>.
2. Australian Institute of Health and Welfare. My Local Area Hospitals. 2019 [Cited January 2020]. Available from:

<https://www.aihw.gov.au/reports-data/myhospitals/my-local-area/hospitals>.

1. Australian Bureau of Statistics. Australian Statistical Geography Standard (ASGS): Volume 5 - Remoteness Structure (cat. no. 1270.0.55.005) 2016 [Cited January 2020]. Available from: <https://www.abs.gov.au/websitedbs/D3310114.nsf/home/remoteness+structure>.
2. Australian Bureau of Statistics. Population Density, Cat. 3218.0 Regional Population Growth, Australia 2016 [Cited December 2019].

<https://www.abs.gov.au/ausstats/abs@.nsf/Previousproducts/3218.0Main%20Features752016>

1. Alessandri LM, Stanley FJ, Waddell VP, Newnham J. Stillbirths in Western Australia 1980-1983: influence of race, residence and place of birth. Aust N Z J Obstet Gynaecol 1988; 28(4): 284-92. Epub 1988/11/01. PubMed PMID: 3250446.
2. Abdel-Latif ME, Bajuk B, Oei J, et al. Does rural or urban residence make a difference to neonatal outcome in premature birth? A regional study in Australia. Arch Dis Child Fetal Neonatal Ed 2006; 91(4): F251-6. doi: 10.1136/adc.2005.090670. PubMed PMID: 16428354; PubMed Central PMCID: PMCPMC2672724.
3. Akl N, Coghlan EA, Nathan EA, Langford SA, Newnham JP. Aeromedical transfer of women at risk of preterm delivery in remote and rural Western Australia: why are there no births in flight? Aust N Z J Obstet Gynaecol 2012; 52(4): 327-33. doi: 10.1111/j.1479-828X.2012.01426.x. PubMed PMID: 22494047.
4. Department of Health, Government of Western Australia. Framework for the care of neonates in Western Australia. Perth: Health Networks Branch, Department of Health, Western Australia; 2009. [Cited December 2019]. Available from: <https://ww2.health.wa.gov.au/~/media/Files/Corporate/general%20documents/Health%20Networks/Womens%20and%20Newborns/Framework-for-the-Care-of-Neonates-in-WA.pdf>.
