# Supplementary material for: Healthcare factors associated with the risk of antepartum and intrapartum stillbirth in migrants in Western Australia (2005-2013): A retrospective cohort study
Source: PLoS Med. 2020 Mar 17;17(3):e1003061. doi: 10.1371/journal.pmed.1003061 (PMC7077810; doi:10.1371/journal.pmed.1003061)
Supplement: S1 RECORD Checklist — (DOCX) [file pmed.1003061.s002.docx]

**The RECORD statement – checklist of items, extended from the STROBE statement, that should be reported in observational studies using routinely collected health data.**

|  | **Item No.** | **STROBE items** | **Location in manuscript where items are reported** | **RECORD items** | | | **Location in manuscript where items are reported** |
| --- | --- | --- | --- | --- | --- | --- | --- |
| **Title and abstract** | | | | | | | |
|  | 1 | (a) Indicate the study’s design with a commonly used term in the title or the abstract (b) Provide in the abstract an informative and balanced summary of what was done and what was found | (a) & (b) addressed in the Methods and Findings section of the abstract. | RECORD 1.1: The type of data used should be specified in the title or abstract. When possible, the name of the databases used should be included.  RECORD 1.2: If applicable, the geographic region and timeframe within which the study took place should be reported in the title or abstract.  RECORD 1.3: If linkage between databases was conducted for the study, this should be clearly stated in the title or abstract. | | | The abstract of the paper, in the Methods and Findings section, includes 1.1, 1.2, and 1.3. |
| **Introduction** | | | | | | | |
| Background rationale | 2 | Explain the scientific background and rationale for the investigation being reported | In the Introduction, the first two paragraphs explain the scientific background and rationale for the investigation. |  | | |  |
| Objectives | 3 | State specific objectives, including any prespecified hypotheses | From paragraph three to the end of the Introduction section, we have discussed the pre-specified hypotheses and objectives of the study: *“we hypothesised that healthcare factors may explain the disparities observed…”* |  | | |  |
| **Methods** | | | | | | | |
| Study Design | 4 | Present key elements of study design early in the paper | Methods, first paragraph | |  |  | |
| Setting | 5 | Describe the setting, locations, and relevant dates, including periods of recruitment, exposure, follow-up, and data collection | Methods section, in first paragraph, explains the setting, location, the period of the study, and the source of data. | |  |  | |
| Participants | 6 | *(a) Cohort study* - Give the eligibility criteria, and the sources and methods of selection of participants. Describe methods of follow-up  *Case-control study* - Give the eligibility criteria, and the sources and methods of case ascertainment and control selection. Give the rationale for the choice of cases and controls  *Cross-sectional study* - Give the eligibility criteria, and the sources and methods of selection of participants  *(b) Cohort study* - For matched studies, give matching criteria and number of exposed and unexposed  *Case-control study* - For matched studies, give matching criteria and the number of controls per case | Methods section, in the first paragraph, explains the population of the study, *“the entire non-Indigenous population of births occurred in WA from 1 January 2005 to 31 December 2013”* | | RECORD 6.1: The methods of study population selection (such as codes or algorithms used to identify subjects) should be listed in detail. If this is not possible, an explanation should be provided.  RECORD 6.2: Any validation studies of the codes or algorithms used to select the population should be referenced. If validation was conducted for this study and not published elsewhere, detailed methods and results should be provided.  RECORD 6.3: If the study involved linkage of databases, consider use of a flow diagram or other graphical display to demonstrate the data linkage process, including the number of individuals with linked data at each stage. | 6.1: Methods section, in the first paragraph. By design, the dataset provided to the researchers included all non-Indigenous births occurred in WA from 1 January 2005 to 31 December 2013.  6.2 & 6.3: Methods section, from the second paragraph onward under the sub-heading “Data Sources and Linkage”. References#11-18 & 78 describe the general linkage processes, quality and validation studies published on the data source. No specific validation for this study was conducted. Reference #6 contains a previous study published by the same authors, from the same project. | |
| Variables | 7 | Clearly define all outcomes, exposures, predictors, potential confounders, and effect modifiers. Give diagnostic criteria, if applicable. | Please see the following sections in Methods:  Exposures  Outcomes  Other variables | | RECORD 7.1: A complete list of codes and algorithms used to classify exposures, outcomes, confounders, and effect modifiers should be provided. If these cannot be reported, an explanation should be provided. | All variables used (original ones from the database or those created by the researchers) have been described in detail in the Methods whilst the data source of the variable is also mentioned. | |
| Data sources/ measurement | 8 | For each variable of interest, give sources of data and details of methods of assessment (measurement).  Describe comparability of assessment methods if there is more than one group | All variables used (original ones from the database or those created by the researchers) have been described in detail in the Methods under sub-headings: Exposures, Outcomes, Other variables. The data source of each variable is also mentioned in the above section. | |  |  | |
| Bias | 9 | Describe any efforts to address potential sources of bias | In Methods, Exposures & Outcomes sections explain cross-source checking from different datasets undertaken to ascertain exposures and outcomes. “Other variables” section describes sub-group analysis for variables that were partially available, exclusion of terminations of pregnancies from the outcomes as well as managing the 3.3% missing data for ARIA and IRSD variables.  “Sensitivity analysis” section also describes efforts to check and address potential sources of bias. | |  |  | |
| Study size | 10 | Explain how the study size was arrived at | Methods, in “Study Design and Participants” describe the population of study and at the final statement of “Outcomes” section describes the exclusions. | |  |  | |
| Quantitative variables | 11 | Explain how quantitative variables were handled in the analyses. If applicable, describe which groupings were chosen, and why | Methods under the “Statistical analysis” section. | |  |  | |
| Statistical methods | 12 | (a) Describe all statistical methods, including those used to control for confounding  (b) Describe any methods used to examine subgroups and interactions  (c) Explain how missing data were addressed  (d) *Cohort study* - If applicable, explain how loss to follow-up was addressed  *Case-control study* - If applicable, explain how matching of cases and controls was addressed  *Cross-sectional study* - If applicable, describe analytical methods taking account of sampling strategy  (e) Describe any sensitivity analyses | For (a) & (b) please see the Methods section under the “Statistical analysis” section.  For (c), please see the Methods section at the end of “Other variables” section where missing data for IRSD and ARIA is addressed.  (d) Loss to follow-up is not applicable to this Cohort study due to the outcomes studied in this paper (stillbirths). | |  |  | |
| Data access and cleaning methods |  | .. |  | | RECORD 12.1: Authors should describe the extent to which the investigators had access to the database population used to create the study population.  RECORD 12.2: Authors should provide information on the data cleaning methods used in the study. | 12.1: In the Methods, paragraph one describes that de-identified data was provided to the investigators by the WA Department of Health via the WA Data Linkage System.  12.2: The details of data sources, cross-source checking for ascertainment/retrieving the missing values, exclusions and new variables created are described in Methods as well. Reference #6 also contains some details on data extraction and cleaning that were previously published. | |
| Linkage |  | .. |  | | RECORD 12.3: State whether the study included person-level, institutional-level, or other data linkage across two or more databases. The methods of linkage and methods of linkage quality evaluation should be provided. | 12.3: in Methods, Data Sources and Linkage section and the references #11-14 describe the data linkage (person-level), quality and reliability of the data linkage procedures, “ *widely known as best practice*”, in detail. | |
| **Results** | | | | | | | |
| Participants | 13 | (a) Report the numbers of individuals at each stage of the study (*e.g.*, numbers potentially eligible, examined for eligibility, confirmed eligible, included in the study, completing follow-up, and analysed)  (b) Give reasons for non-participation at each stage.  (c) Consider use of a flow diagram | (a) & (b): Please see Results, first paragraph.    (c) : N/A | RECORD 13.1: Describe in detail the selection of the persons included in the study (*i.e.,* study population selection) including filtering based on data quality, data availability and linkage. The selection of included persons can be described in the text and/or by means of the study flow diagram. | | | 13.1 Please see Methods, first paragraph, Study Design and Participants section. Also, Results, first paragraph. |
| Descriptive data | 14 | (a) Give characteristics of study participants (*e.g.*, demographic, clinical, social) and information on exposures and potential confounders  (b) Indicate the number of participants with missing data for each variable of interest  (c) *Cohort study* - summarise follow-up time (*e.g.*, average and total amount) | (a) Please see Results, first paragraph.  (b) Methods, Exposures, Outcomes, Other variables.  (c) not applicable as the outcomes are live/stillbirths |  | | |  |
| Outcome data | 15 | *Cohort study* - Report numbers of outcome events or summary measures over time  *Case-control study* - Report numbers in each exposure category, or summary measures of exposure  *Cross-sectional study* - Report numbers of outcome events or summary measures | Please see Results section including tables 2-4 |  | | |  |
| Main results | 16 | (a) Give unadjusted estimates and, if applicable, confounder-adjusted estimates and their precision (e.g., 95% confidence interval). Make clear which confounders were adjusted for and why they were included  (b) Report category boundaries when continuous variables were categorized  (c) If relevant, consider translating estimates of relative risk into absolute risk for a meaningful time period | (a) Please see Results section including tables 3-5.  (b) Please see Results, tables 1 & 2.  (c) Please see the Results, cumulative incidence rates reported in the tables 1 & 2. |  | | |  |
| Other analyses | 17 | Report other analyses done—e.g., analyses of subgroups and interactions, and sensitivity analyses | Please see the Results, sections: Antenatal care, Birth-attendant and intrapartum care, Interpreter service, Controlling for the effect of LBW and PTB, Private health Insurance and Sensitivity analysis |  | | |  |
| **Discussion** | | | | | | | |
| Key results | 18 | Summarise key results with reference to study objectives | Key results have been summarised in the Discussion |  | | |  |
| Limitations | 19 | Discuss limitations of the study, taking into account sources of potential bias or imprecision. Discuss both direction and magnitude of any potential bias | Please see the Discussion, Limitations section. | RECORD 19.1: Discuss the implications of using data that were not created or collected to answer the specific research question(s). Include discussion of misclassification bias, unmeasured confounding, missing data, and changing eligibility over time, as they pertain to the study being reported. | | | 19.1: In the Limitation section non-independence bias, misclassification, residual confounding due to variables not available, effect of change in standards over time, and missing data have all been included. |
| Interpretation | 20 | Give a cautious overall interpretation of results considering objectives, limitations, multiplicity of analyses, results from similar studies, and other relevant evidence | Please see the Discussion, the Limitations section and the final sentence which reads:  *“Considering all the above, the results should be interpreted with caution.”* |  | | |  |
| Generalisability | 21 | Discuss the generalisability (external validity) of the study results | Please see the Generalisability section in the Discussion. |  | | |  |
| **Other Information** | | | | | | | |
| Funding | 22 | Give the source of funding and the role of the funders for the present study and, if applicable, for the original study on which the present article is based | Financial disclosure statement. |  | | |  |
| Accessibility of protocol, raw data, and programming code |  | .. |  | RECORD 22.1: Authors should provide information on how to access any supplemental information such as the study protocol, raw data, or programming code. | | | Data availability statement. |

*Reference: Benchimol EI, Smeeth L, Guttmann A, Harron K, Moher D, Petersen I, Sørensen HT, von Elm E, Langan SM, the RECORD Working Committee. The REporting of studies Conducted using Observational Routinely-collected health Data (RECORD) Statement. *PLoS Medicine* 2015; in press.

*Checklist is protected under Creative Commons Attribution ([CC BY](http://creativecommons.org/licenses/by/4.0/)) license.
